# Numerical Deformation Analysis of Reinforced Lightweight Aggregate Concrete Flexural Members

**DOI:** 10.3390/ma15031005

**Published:** 2022-01-27

**Authors:** Darius Bacinskas, Deividas Rumsys, Gintaris Kaklauskas

**Affiliations:** Department of Reinforced Concrete Structures and Geotechnical Engineering, Faculty of Civil Engineering, Vilnius Gediminas Technical University—Vilnius TECH, 10223 Vilnius, Lithuania; deividas.rumsys@gmail.com (D.R.); gintaris.kaklauskas@vilniustech.lt (G.K.)

**Keywords:** lightweight aggregate concrete, reinforced concrete, slab, bridge girder, curvature, short-term loading, tension stiffening, constitutive model, numerical modelling

## Abstract

In the modern construction industry, lightweight aggregate concrete (LWAC) is often used to produce load-bearing structural members. LWAC can be up to 40% lighter by volume than normal strength concrete. However, the lack of adequate numerical models often limits the practical application of innovative building materials such as lightweight concrete in real projects. The present study conducted a comparative numerical deformation analysis of a full-scale bridge deck slab and girder. Using the physical model proposed by the authors and the finite element software ATENA, the deformations of full-scale lightweight and traditional reinforced concrete elements under the short-term effects of permanent and variable loads were compared. Depending on the safety and serviceability limit requirements, it was found that the amount of longitudinal reinforcement in lightweight reinforced concrete elements could be reduced compared with that in standard reinforced concrete elements with the same parameters. The results of the numerical analysis showed that the deformation analysis model proposed by the authors could serve as an alternative tool for the design of lightweight concrete flexural members with the selection of optimum geometric and reinforcement parameters limited by the stiffness condition.

## 1. Introduction

In the modern construction industry, lightweight aggregate concrete (LWAC), which can be up to 40% lighter than standard strength concrete by volume, is often used in the production of load-bearing structures in addition to standard strength concrete. For lightweight structural concrete, natural or artificial lightweight aggregates are used in addition to heavy aggregates (sand, gravel, crushed stone) [1]. Artificial aggregates also include secondary raw materials, increasingly used in concrete mixes in recent years [2]. The use of lightweight structural concrete for structural members has many advantages, such as a reduced amount of required reinforcement, smaller element cross sections, the possibility of constructing higher-rise buildings, and lower material costs for the installation of foundations [3,4]. These advantages also reduce the overall cost of the building. The use of lightweight concrete instead of standard concrete can improve the performance of structures. Lightweight concrete has better thermal and acoustic insulation properties than standard concrete, which significantly reduces the energy consumption in buildings constructed of lightweight concrete during their operation [5,6,7,8]. Real et al. [9] found that the use of lightweight concrete instead of standard concrete in the load-bearing structures of European buildings would result in a 15% reduction in thermal energy consumption.

In addition to these advantages, lightweight concrete has better durability properties [10] than standard concrete, including higher frost resistance, fire resistance, and seismic loading. This has led to the widespread use of lightweight concrete in many countries to construct high-span bridge decks [11] and multistory buildings [12]. On the other hand, the increasing design requirements for advanced concrete structures, increasing intensity of external mechanical effects, and aggressiveness of the surrounding environment have highlighted the need for adequate serviceability models for the analysis of reinforced concrete (RC) structures. RC is an exceptional structural material for two reasons: the extent of its practical use and the complexity of its mechanical behaviour. The latter characteristic is attributed to phenomena such as concrete cracking, shrinkage and creep effects, and tension stiffening [13]. The relatively low tensile strength and cracking resistance of concrete can be identified as major structural concerns.

A large number of analytical and numerical techniques for the serviceability analysis of traditional RC elements have been proposed and presented to the engineering community [14,15,16]. However, relatively few universal approaches are dedicated to the deformational analysis of reinforced LWAC members [1].

The authors recently proposed a constitutive model for assessing flexural stiffening of reinforced LWAC beams subjected to short-term loading [17]. Further research has included a comparative analysis of the curvature calculations using analytical code methods EN1992-1 [18] and ACI 318-19 [19], as well as a numerical analysis using the constitutive model for cracked tensile lightweight concrete proposed by the authors [20]. The analysis was carried out using experimental data published in the literature for 51 lightweight RC members obtained under five experimental programs. A comparison of the theoretical and experimental results showed that the most accurate predictions were obtained using numerical analysis and the constitutive model proposed by the authors.

It should be noted that the lack of adequate numerical models often limits the practical application of innovative building materials such as lightweight concrete in real projects [21]. This is due to the resulting uncertainties in standard design methods and calculation errors, which are generally unacceptable to civil engineers in terms of safety and reliability. In this study, a comparative numerical deformation analysis of a full-scale bridge deck slab and girder was conducted. Using the physical model proposed by the authors and the finite element software ATENA, the deformations of full-scale lightweight and traditional RC elements under the short-term effects of permanent and variable loads were compared. Depending on the safety and serviceability limit requirements, it was found that the amount of longitudinal reinforcement in lightweight RC elements could be reduced compared with standard RC elements with the same parameters. The results of the numerical analysis showed that the deformation analysis model proposed by the authors is an alternative tool for the design of lightweight concrete flexural members with the selection of optimum geometric and reinforcement parameters limited by the stiffness condition.

## 2. Numerical Model for Finite Element Analysis of LWAC Flexural Members

**Figure 1:**
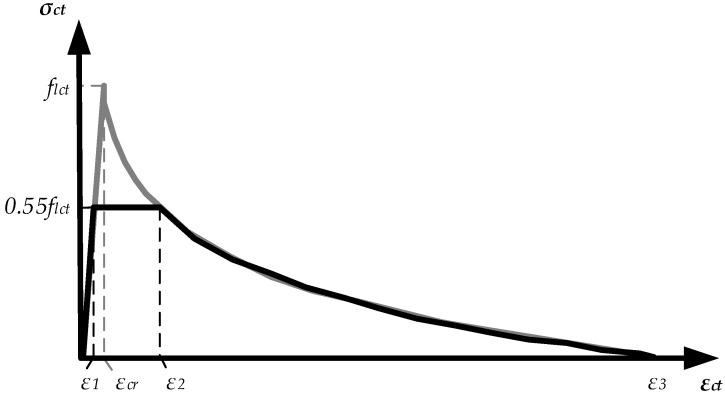
Tension–stiffening model of structural lightweight tensile concrete [1].

The numerical inverse technique used to derive the constitutive model incorporating the experimental results of reinforced lightweight concrete elements is discussed in detail in references [1,19,20]. The fundamental aspects of physical modelling are described below. The methodology is based on a layered section model, which implies the successive application of direct (curvature prediction) and inverse (constitutive modelling) approaches. The method proposed by Kaklauskas and Ghaboussi [22] was applied to the constitutive modelling to obtain the average stress and average strain diagrams for cracked tensile concrete. As a result, tension–stiffening diagrams with eliminated shrinkage [23,24] were obtained. The proposed model (Figure 1) is approximated as a three-curve diagram. The ascending branch of the curve represents the elastic behaviour of the RC before cracking. Meanwhile, the horizontal and descending branches characterise the stages of crack formation and further development, respectively. The tensile strength is *σ_ct_* = 0.55*f_lct_*, where *f_lct_* is the average tensile strength of LWAC calculated according to the Eurocode 2 (EC2) standard [18].

Strain *ε*_1_ is calculated as follows:

(1) $$\varepsilon_{1}=0.55\varepsilon_{cr},$$

where *ε_cr_* = *f_lct_*/*E_lcm_* is the theoretical cracking strain corresponding to the tensile strength, and *E_lcm_* is the modulus of elasticity of concrete calculated according to EC2 depending on the compressive strength of the concrete.

The descending branch of the diagram is approximated by the following relationship:

(2) $$\sigma_{ct}=f_{lct}\left(1-0.27\ln\left(\frac{\varepsilon_{ct}}{\varepsilon_{cr}}\right)-0.21\rho_{R}\right),$$

where ρ*_R_* is the reinforcement percentage [%].

Strain *ε*_2_ is calculated as follows:

(3) $$\varepsilon_{2}=\varepsilon_{cr}e^{1.667-0.78\rho_{R}}.$$

The length of the descending branch is defined by the maximal strain, *ε*_3_, corresponding to zero stress. This strain is calculated as follows:

(4) $$\varepsilon_{3}=\varepsilon_{cr}e^{3.7-0.78\rho_{R}}.$$

**Figure 2:**
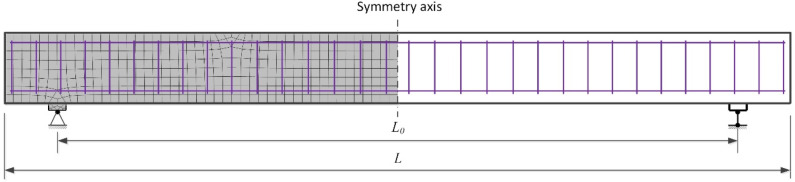
A typical finite element model and support conditions of reinforced LWAC flexural member. *L*_0_ is the span; *L* is the total length.

Nonlinear numerical analysis was performed using the finite element software ATENA (version: 5.7.0, Prague, Czech Republic). In most cases, two-dimensional finite element models of RC elements were created by employing constitutive models of compressive and tensile concrete and the reinforcement. The behaviour of the reinforcement was represented by an elastic–plastic model corresponding to the yield strength of steel and the modulus of elasticity. A linear elastic diagram was used to model the compressive concrete. Justification is that the stress–strain relation in compression for LWAC adopted in EC2 is very close to linear. Plastic deformations occur only at high levels of compressive stresses. The difference in deflection estimates at service load when linear and nonlinear diagrams of compressive concrete were assumed are marginal. The proposed constitutive model (Figure 1) was used to describe the behaviour of the LWAC in tension. The 3D Nonlinear Cementitious 2 User material model (based on the SBETA material model in ATENA) was utilised. The concrete without cracks was considered an isotropic body, and concrete with cracks was considered an orthotropic body. The smeared crack and fracture mechanics approaches were combined in ATENA to assess the nonlinear behaviour of RC elements after cracking. In this study, a fixed crack model was used. The fracture mechanics approach employed in ATENA for softening behaviour is based on the crack band model. Isoparametric quadrilateral finite elements with eight degrees of freedom and four integration points were used to model the concrete beams. The reinforcement bars were modelled using truss finite elements. A typical finite element model including the support conditions of the RC member is presented in Figure 2.

## 3. Accuracy Assessment of the Numerical Finite Element Model

A comparative numerical analysis was carried out for three full-scale experimental panels published by Vakhshouri [25] to evaluate the proposed numerical finite element model. The experimental program consisted of testing three full-scale RC slabs under short-term loading. All specimens had rectangular sections with a nominal length of 3800 mm, a span of 3500 mm, a depth of 161 mm, and a width of 400 mm. All beams were tested by applying a uniformly distributed load using concrete blocks. Two different loading levels were selected for the tests. Experimental loads were applied at the age of 14 days. The tensile reinforcement consisted of four 12-mm-diameter bars, ensuring a reinforcement percentage of 0.83%. The concrete cover from the centroid of the longitudinal reinforcement to the nearest concrete surface was 25 mm. The specimens were cast using a concrete mix with the following physical and mechanical parameters: a density of 2000 kg/m^3^, compressive strength of 31 MPa, and tensile strength of 2.29 MPa.

**Figure 3:**
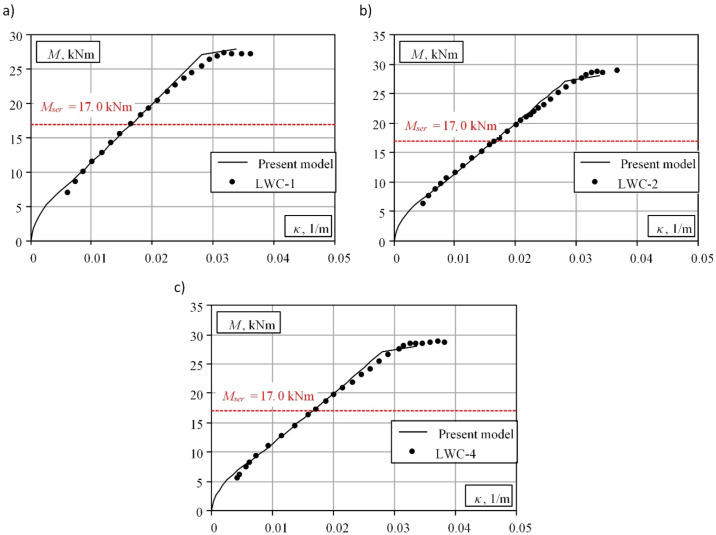
Comparison of theoretical and experimental moment–curvature diagrams: (**a**) slab LWC-1; (**b**) slab LWC-2; (**c**) slab LWC-3. *M_ser_* is a bending moment corresponding to the service load.

A comparison of the numerical and experimental bending moment–curvature diagrams is shown in Figure 3. The presented comparison shows that the numerical model of reinforced lightweight concrete proposed by the authors is suitable for the deformation analysis of full-scale structures. The theoretical model provides satisfactory prediction results at the stages preceding reinforcement yielding. The maximum discrepancy between the experimental and theoretical deflections at the service load (*M_ser_*) does not exceed 2.5%. The analysis of the obtained results shows that the authors’ proposed model can be considered an alternative tool for analysing the real stress–strain state of lightweight RC.

## 4. Numerical Modelling of Full-Scale Reinforced LWAC Flexural Members

This section describes the comparative numerical deformation analysis of a full-scale bridge deck slab and girder under short-term loading. The behaviours of full-scale lightweight and standard RC structural elements under the short-term effects of permanent and variable loading were compared using the proposed physical model and the finite element program ATENA. Depending on the safety and serviceability limit requirements, it was determined that the amount of longitudinal reinforcement could be reduced in lightweight RC elements compared with standard RC elements with the same parameters. The subject of this study is a free-supported standard and lightweight concrete bridge deck slab and girder designed according to the EC2 standard [18]. The slab was designed for two different levels of characteristic variable loads, which represent the minimum and maximum values of pedestrian loads 2.5 kPa and 5.0 kPa, respectively, specified in EC1 [26]. The slab length was 6.3 m, the span was 6.0 m, and the width was 1 m. The height of the slab (0.3 m) was chosen considering the operating variable load and the deflection limit recommendations provided in the EC2 standard [18]. The protective concrete layer was 40 mm thick. The density of the standard concrete was 2300 kg/m^3^, and the density of the lightweight concrete was 1800 kg/m^3^. The characteristic permanent load was calculated by considering the slab’s self-weight (dead load) and deck load (10.6 kPa and 9.1 kPa for standard and lightweight concrete, respectively).

**Figure 4:**
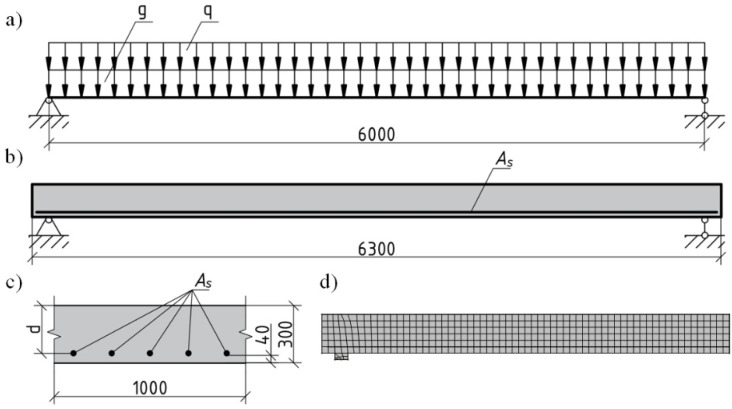
RC slab details [1]: (**a**) static scheme; (**b**) side view; (**c**) cross section; (**d**) finite element model (dimensions are in mm). g is the dead load; q is the live pedestrians load; *A_s_* is the area of reinforcement.

C30/37 concrete was used for the normal RC elements, and LC30/33 lightweight concrete with the same compressive strength was used for the lightweight concrete. An S500 longitudinal reinforcement was used to reinforce both elements. Transverse reinforcements were not utilised. The normal and lightweight concrete slabs were designed to ensure mechanical resistance in flexure and shear for the designated initial parameters. According to the calculated safety limit state, normal RC slabs subjected to variable loads of 2.5 kPa and 5.0 kPa were reinforced with 5∅14 mm (ρ*_R_* = 0.3%) and 5∅16 mm (ρ*_R_* = 0.4%) diameter bars, respectively. The behaviour of the LWAC slabs with a reduced reinforcement intensity of 6∅12 mm (*ρ_R_* = 0.27%) and 8∅12 mm (*ρ_R_* = 0.36%) was also investigated to reveal the advantages of lightweight concrete and the reduced permanent load. The shrinkage deformations of concrete before loading were calculated as −1 × 10^–4^ and –1.14 × 10^–4^ for normal and lightweight concrete, respectively. The free shrinkage strain was assessed according to EC2 [18], assuming the following characteristics: *t_s_* = 7 days—age of concrete at the end of curing, *t*_0_ = 28 days—concrete age at loading, RH = 75%—relative humidity, CEM 42.5 N—cement type. Numerical modelling was performed using the finite element program ATENA by applying the principles presented in Section 2. The latter section also includes the material models for LWAC. For ordinary concrete, a nonlinear stress–strain diagram suggested by EC2 [18] was adopted for compression, whereas the tension stiffening model proposed by Sokolov [27] was used for simulating the tensile behaviour. The computational scheme of the slab, main parameters, and finite element model is shown in Figure 4.

**Figure 5:**
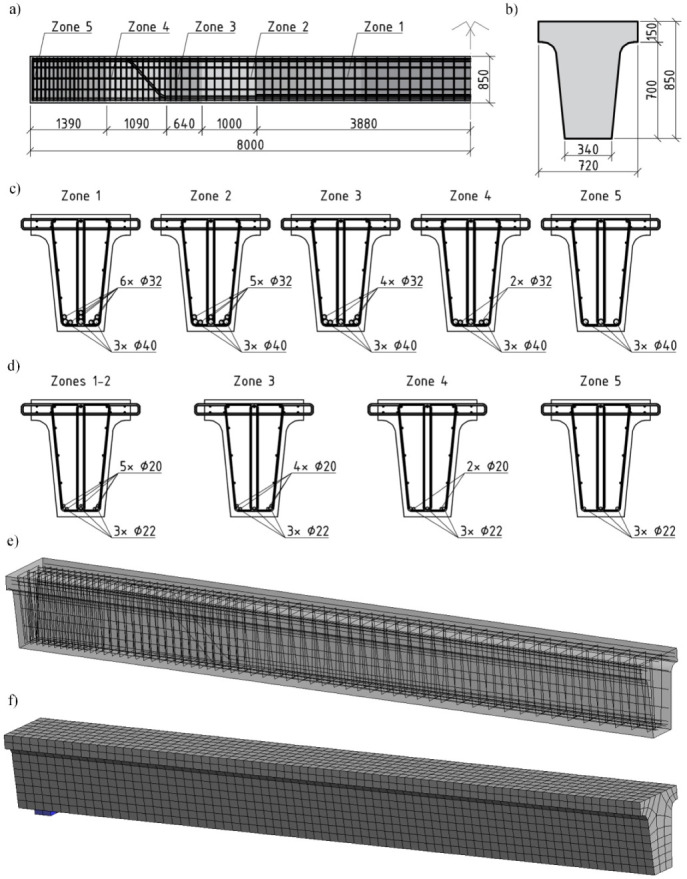
Reinforced concrete bridge girders used in the analysis [1]: (**a**) longitudinal cross section and division into reinforcement zones; (**b**) cross section; (**c**) reinforcement details of the roadway bridge girder in different zones; (**d**) reinforcement details of the footbridge girder in different zones; (**e**) computer model; (**f**) finite element model (dimensions are in mm).

The modelling of the bridge deck girder was based on similar principles. A typical girder with a length of 16 m and a T-shaped cross section with a 15.4 m design span used for roadway and pedestrian bridge decks was analysed. The longitudinal and transverse sections of the girder are shown in Figure 5a,b. The girder was divided into five distinct sections based on the number and arrangement of the longitudinal tension reinforcement. The reinforcement of each section for the roadway and pedestrian bridge girders is shown in Figure 5a,c,d.

The roadway bridge deck girder was made of C35/45 normal weight concrete (NWC) and reinforced with S500 reinforcing bars. The girder was designed to withstand a design bending moment of *M_Rd_* = 2140 kNm. The characteristic bending moment due to the self-weight of the girder and the permanent load on the bridge deck was *M_gk_* = 476 kNm. The percentage of reinforcement in the middle section of the girder was 2.33%. LC35/38 concrete with density *ρ* = 1900 kg/m^3^ was used for the lightweight concrete beams with the corresponding geometric parameters and reinforcement scheme. Owing to the reduced self-weight, the characteristic bending moment under the permanent load for these girders decreased to *M_gk_* = 407 kNm.

The pedestrian bridge girder was designed for a live load of 5 kPa. C30/37 concrete and S500 reinforcements were used for the reinforced NWC beams. The longitudinal reinforcement of the girders in different sections (Figure 5d) was chosen to ensure the load-bearing capacity of the standard section. LC30/33 lightweight concrete with density *ρ* = 1800 kg/m^3^ was used for the lightweight concrete beams. The characteristic bending moment caused by permanent loading due to the lower self-weight of the girder decreased from *M_gk_* = 459 kNm to *M_gk_* = 389 kNm. The behaviour of the NWC and LWAC beams with the same reinforcement intensity was compared in the first stage for the lightweight and standard concrete elements. Taking into account the reduced permanent load, the behaviour of the LWAC beam in the middle zone (Figure 5d, zones 1–2) was further investigated in the second stage by reducing the reinforcement percentage from 0.72% to 0.64%. Thus, the diameter of the longitudinal reinforcement of the girder examined in the second stage was changed from Ø20 mm to Ø18 mm.

The influence of shrinkage strains, *ε_cs_*, in the stage prior to loading was evaluated using numerical analysis. The values of *ε_cs_* were calculated by EC2 [18] similarly to the bridge slab, assuming different section and concrete grade characteristics. For the roadway RC bridge girders, shrinkage strain values of −5.8 × 10^−5^ and −6.2 × 10^–5^ were obtained for standard and lightweight concrete, respectively. The pedestrian bridge girders’ shrinkage strain values were −5.4 × 10^−5^ for standard concrete and −5.8 × 10^−5^ for lightweight concrete. The GID program module integrated into the finite element program ATENA was used to model the girders. The numerical model was developed using three-dimensional isoparametric finite elements with 24 degrees of freedom and eight integration points. The other aspects of the modelling were the same as those used in the numerical modelling of the girders. The computer-simulated girder image is shown in Figure 5e, and the division into finite elements is shown in Figure 5f. It should be noted that owing to the uncertainties related to the prediction of the shear strength of lightweight concrete, the shear strength of the latter elements has not been assessed in the analysis presented in this section. It was assumed that the strengths of the standard and lightweight concrete elements were equal. The sufficient shear strength of the reinforced lightweight concrete elements was confirmed by the simulation results: all elements decomposed in the standard section owing to the bending moment effect.

## 5. Numerical Modelling Results

**Figure 6:**
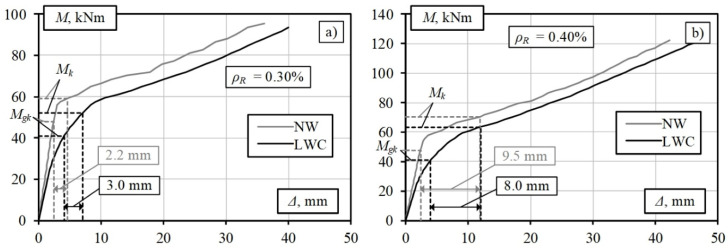
Results of the numerical modelling of slabs subjected to variable loads: (**a**) 2.5 kPa; (**b**) 5.0 kPa.

The simulation results for the standard and lightweight concrete slabs with the same parameters are shown in Figure 6. The results were compared at the total characteristic bending moment, *M_Ek_*. In bridge design practice, deflections caused by permanent loads, including long-term changes, are usually compensated by the initial deflection of the structures. The stiffness of structures is usually limited by deflections caused by characteristic variable traffic loads, which in turn are limited by the relative deflection limits regulated by the design standards. The limitation of deflections in lightweight RC owing to its lower stiffness can be a decisive factor in the design of these structures.

Figure 6 shows that the deflection of the LWAC slab with a reinforcement percentage of 0.30% due to the variable load (Δ*_qk_* = 3.0 mm) is slightly higher than that of the standard RC slab (Δ*_qk_* = 2.2 mm). In general, the deflection values for both elements are small. Slightly different trends are obtained for slabs with a reinforcement percentage of 0.40% (Figure 6). The deflection of the standard RC slab due to the variable load is higher than that of the LWAC slab: 9.5 mm and 8.0 mm, respectively. These differences can be explained by the lower total bending moment of the LWAC slab due to the lower permanent load. The detailed calculation results are summarised in Table 1, where *M_gk_* is the bending moment due to the characteristic permanent load, *M_Ek_* is the bending moment due to the characteristic total load, *M_Ed_* is the bending moment due to the design total load, *M_Rk_* is the resistance characteristic bending moment of the element, and Δ*_qk_* is the deflection due to the characteristic variable load.

The lower weight of the LWAC slabs can reduce the required amount of longitudinal reinforcement compared with slabs made of standard concrete with the same parameters. However, the reduced stiffness of the elements, in this case, may require consideration of the deflection constraints. These values are regulated by various design standards and depend on the structure’s purpose.

**Figure 7:**
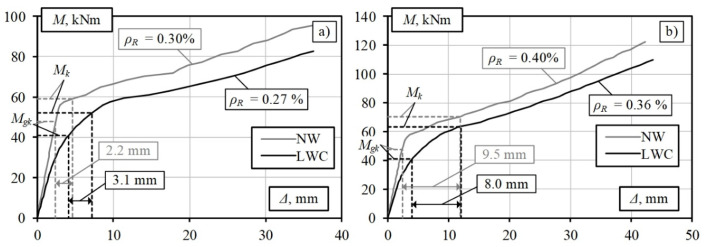
Modelling results for LWAC slabs with reduced reinforcement ratio with the variable load being (**a**) 2.5 kPa and (**b**) 5.0 kPa.

The results of the analysis of the LWAC slabs with a reduced reinforcement percentage are shown in Figure 7. The results show a slight increase in the deflection value for the slab with a reinforcement percentage of 0.27%: from 3.0 mm (for the LWAC slab where *ρ_R_* = 0.3%) to 3.1 mm, which is acceptable from a practical perspective. Thus, the amount of longitudinal reinforcement can be reduced by 12% by designing an LWAC bridge deck slab subjected to a variable pedestrian load of 2.5 kPa. A similar trend is observed for the slab subjected to a load of 5 kPa (Figure 7b). The resulting deflection values remain almost unchanged, and the amount of longitudinal reinforcement is reduced by 10%. The results show that the higher the ratio between the moments caused by the permanent load (*M_gk_*) and total load (*M_Ek_*), the higher the efficiency of using LWAC in load-bearing structures will be, which leads to a more significant reduction in the amount of longitudinal reinforcement. The values of these ratios for the deck slabs are listed in Table 1.

**Figure 8:**
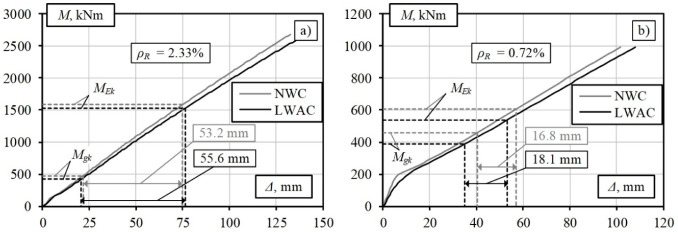
Modelling results for bridge girders [1]: (**a**) roadway bridge; (**b**) pedestrian bridge.

The modelling results for the roadway bridge girders are presented in Figure 8a, and those for the pedestrian bridge are presented in Figure 8b. Figure 8a shows that at a high reinforcement percentage (*ρ_R_* = 2.33%), the numerical deformation analyses of elements having the same parameters using the LWAC and NWC physical models yield approximately the same calculation results. The slight differences in the curves for the LWAC and NWC girders are related to the different moduli of elasticity of these materials. The LWAC and NWC results are also in good agreement because the effect of tensile concrete between the cracks on the stiffness in highly reinforced elements is insignificant. Owing to this factor and the low ratio of permanent to total load (0.28–0.30, Table 2), the reduction in concrete density does not have a significant structural effect.

**Figure 9:**
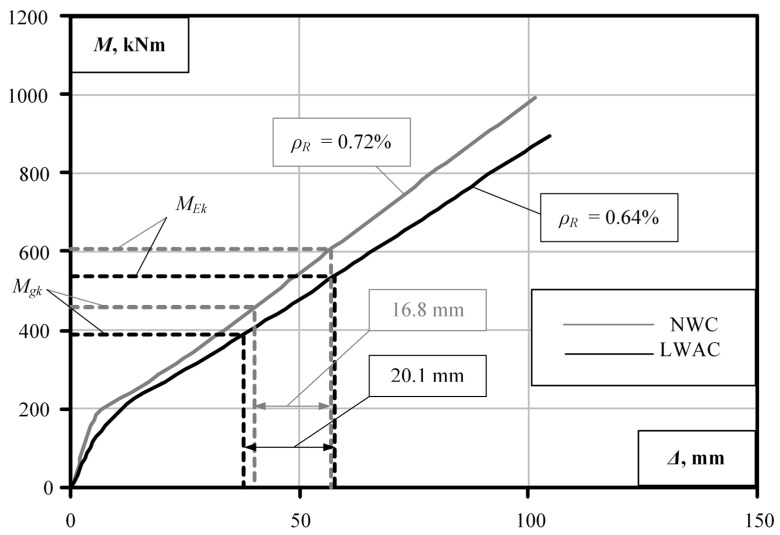
Results of the analysis of bridge girders with a reduced reinforcement ratio.

The opposite trend is observed for the pedestrian bridge deck girder (Figure 8b). The ratios of the permanent to a total load of these girders are 0.72–0.76 (Table 2). Similar deflection values of 16.8 mm and 18.1 mm are obtained for the standard and lightweight concrete girders, respectively, under variable pedestrian loading. The permanent load level and the deflection values were obtained to allow for a reduction in the intensity of the longitudinal reinforcement of the girders. The numerical modelling results for the LWAC girder of the pedestrian bridge with reduced reinforcement are shown in Figure 9. The deflection value increases from 18.1 mm to 20.1 mm, with the decrease in the reinforcement percentage of the LWAC girder from 0.72% to 0.64%. Considering the length of the element span, the obtained deflection value is practically acceptable. In this case, the amount of reinforcement required is reduced by 11%. The numerical analysis results show that the deformation analysis model proposed by the authors can be considered an alternative tool for the design of lightweight concrete flexural members with the selection of optimum geometric and reinforcement parameters limited by the stiffness condition.

## 6. Conclusions

The numerical finite element deformation analysis of reinforced standard and lightweight aggregate concrete full-scale flexural members yielded the following conclusions.

1. Advanced structural LWAC is a promising innovative material, and its use in structures allows for reductions in the amount of reinforcement required and the cross sections of elements. However, the lack of adequate numerical models often limits the practical application of innovative building materials such as lightweight concrete in real projects.
2. The adequacy of the tension stiffening model for LWAC recently proposed by the authors was verified by comparing theoretical and experimental results for three full-scale lightweight concrete slabs. The comparative analysis showed that satisfactory prediction results were obtained at all loading stages preceding the yielding of reinforcement. The maximum discrepancy between the experimental and theoretical results at service load (*M_Ek_*) did not exceed 2.5%.
3. This study presents the practical application aspects of the proposed model. A full-scale bridge deck slab and girder were chosen as the object of the study. A numerical deformation analysis was performed for these elements. Using the physical model proposed by the authors and the finite element program ATENA, the deformations of full-scale lightweight and conventional RC structural members under the short-term effects of permanent and variable loads were compared.
4. The results showed that similar deflection values were obtained for lightweight and standard concrete elements under the same levels of variable loads. However, in some cases, standard RC elements exhibited higher deflections under variable loading than lightweight concrete members with the same parameters. For example, the deflection obtained in this study for a standard concrete slab with a reinforcement percentage of 0.40% under variable loading was 19% higher than that obtained for a lightweight concrete slab with the same parameters. These differences can be explained by the lower total bending moment of the LWAC slabs due to their lower permanent load.
5. The results revealed that longitudinal reinforcement could be reduced in lightweight RC elements due to the reduced permanent load (up to 12% in the present study) compared with traditional RC elements with the same parameters.
6. The effectiveness of the application of lightweight concrete increases with the ratio of the moments caused by the permanent and total loads. However, the reduced stiffness of the elements may require consideration of deflection limitations. These values are regulated by various design standards and depend on the purpose of the structure.
7. Lightweight concrete is less efficient than standard concrete, with a decrease in the ratio of permanent to total moments and an increase in the reinforcement percentage. In heavily reinforced elements (*ρ_R_* > 2%), the effect of the tensile concrete between cracks on the stiffness is insignificant. In this case, the reduction in the concrete density does not have a significant structural effect.
8. The results of the numerical analysis show that the deformation analysis model proposed by the authors can serve as an alternative tool for the design of lightweight concrete flexural members with a selection of the optimum geometric and reinforcement parameters, which are limited by the stiffness condition. The proposed model can be applied to the analysis of real lightweight concrete elements by implementing the performance-based design concept provided in modern design standards.

## Figures and Tables

**Table 1:** Analysis of the results for the designed slabs.

No.	Concrete Type	*ρ_R_*, %	Bending Moments, kNm					Deflection Δ*_qk_*, mm
			*M_gk_*	*M_Ek_*	*M_gk_*/*M_Ek_*	*M_Ed_*	*M_Rk_*	
1	NWC	0.30	47.7	59.0	0.81	79.6	95.1	2.2
2	LWAC		41.0	52.3	0.78	70.5		3.0
3	NWC	0.40	47.7	70.2	0.68	94.8	122.8	9.5
4	LWAC		41.0	63.5	0.65	85.7		8.0
5	LWAC	0.27	41.0	52.3	0.78	70.5	84.4	3.1
6	LWAC	0.36	41.0	63.5	0.65	85.7	111.7	8.0

**Table 2:** Analysis of the results for the designed girders.

No.	Concrete Type	*ρ_R_*, %	Bending Moments, kNm					Deflection Δ*_qk_*, mm
			*M_gk_*	*M_Ek_*	*M_gk_*/*M_Ek_*	*M_Ed_*	*M_Rk_*	
1	NWC	2.33	476	1585	0.30	2140	2669	53.2
2	LWAC		421	1530	0.28	2066		55.6
3	NWC	0.72	459	607	0.76	819	991	16.8
4	LWAC		389	537	0.72	725		18.1
5	LWAC	0.64	389	537	0.72	725	890	20.1

## Data Availability

The data presented in this study are available on request from the corresponding author.
